# The Six-Year-Old Molars

**Published:** 1888-07

**Authors:** 


### "The Six-Year-Old Molars."

[To the Editor of the "Journal of the British Dental Association."]

*Dear Sir*:—Having read Mr. Bowden's interesting paper on "The Treatment of the Six-Year-Old Molars," in yonr last issue, I thought it might be useful if I mentioned another plan of treating these six-year-old molars; for example, patients under ten are frequently brought to the dentist with these four molars decayed, with a superficial caries confined to almost the whole of the masticating surface; what is the treatment in these cases? We know well that cutting into the dentine is both tedious and painful to the little patients, and at the same time, the pulp cavities being extremely large at this early age, one is always liable to expose the pulp tissue (although the greatest care may have been taken). My plan of treatment in the majority of these cases is as follows: it is most simple, and many will say most unscientific. I cleanse the carious cavities with iodine, and then swab out well with rectified spirit until the carious tissue appears quite dry, and as a rule this causes no pain; I seldom use an excavator or any other instrument save the dressing forceps; I now insert the Sullivan's amalgam, and leave the rest of the treatment to nature. My theory is that by leaving the carious matter alone, one leaves a natural non-conducting pad between the pulp cavity and the metal filling; however unscientific the above mode of treatment may appear, practice proves the opposite. Believe me, dear Sir, faithfully yours,

JAMES RYMER, M.R.C.S., L.D.S.Eng.

Maidstone, May 23rd, 1888.

The plan of treatment of the first permanent molars, above suggested, is to say the least out of the usual course, and quite at variance with the teaching of the past; it should be kept in mind however that the treatment here suggested, is proposed only for a certain class of extreme cases.

We do not here refer to this treatment with the view of criticising; but the question will naturally arise, If this is a good method for these extreme cases, is it not as good or better, for many that are not so extreme, or unfavorable?

But the object of this note is to direct attention to the importance of thorough desiccation, in the preparation of decayed teeth for filling. All careful operators, and close observers, are well aware of the fact that absolute dryness greatly mitigates the intensity of sensitiveness, that so generally attends decay of the teeth; indeed absolute dryness in many cases gives entire relief. The common method of drying out with spunk or bibulous paper will not give this result, but after this the current of heated air, thrown upon the affected surface, is required. The usual method of applying heated air for this porpose is quite defective; that is by the use of the ordinary warm air blow-pipe. With this the current is very irregular and the degree of heat is constantly varying, or at least it diminishes rapidly, whereas it should be kept uniform, or perhaps increased rather than lessened. If, as should always be the case, the saliva is wholly excluded by the rubber dam, there is no difficulty in determining, by the appearance of the surface when absolute dryness has been attained.

In brief, the objects secured by this treatment are

1st. A lessening of the abnormal senstiveness.

2nd. Enabling the operator to proceed with more precision and satisfaction with his work; better results can be attained in working on a dry than on a moist surface.

3rd. Decay is much less likely to return in a dry than in a moist tissue; indeed in the abscence of moisture the ordinary decompositions do not at all take place, and micro-organisms are inoperative, even if they could exist.

A better appliance than has yet been attained should be devised and made available to the profession. Some very expensive and unwieldy affairs have been suggested. But effectiveness, cheapness and simylicity have not yet been combined to a satisfactory degree.

